# Daily 1 km terrain resolving maps of surface fine particulate matter for the western United States 2003–2021

**DOI:** 10.1038/s41597-022-01488-y

**Published:** 2022-08-02

**Authors:** Alan Swanson, Zachary A. Holden, Jon Graham, D. Allen Warren, Curtis Noonan, Erin Landguth

**Affiliations:** 1 Center for Population Health Research, School of Public and Community Health Sciences, University of Montana, 32 Campus Drive, Missoula, MT 59812 USA; 2 US Forest Service Northern Region, Missoula, MT 59807 USA; 3 Mathematical Sciences, University of Montana, 32 Campus Drive, Missoula, MT 59812 USA

**Keywords:** Atmospheric chemistry, Natural hazards, Atmospheric chemistry, Risk factors, Outcomes research

## Abstract

We developed daily maps of surface fine particulate matter (PM_2.5_) for the western United States. We used geographically weighted regression fit to air quality station observations with Moderate Resolution Imaging Spectroradiometer (MODIS) aerosol optical depth (AOD) data, and meteorological data to produce daily 1-kilometer resolution PM_2.5_ concentration estimates from 2003–2020. To account for impacts of stagnant air and inversions, we included estimates of inversion strength based on meteorological conditions, and inversion potential based on human activities and local topography. Model accuracy based on cross-validation was *R*^2^ = 0.66. AOD data improve the model in summer and fall during periods of high wildfire activity while the stagnation terms capture the spatial and temporal dynamics of PM_2.5_ in mountain valleys, particularly during winter. These data can be used to explore exposure and health outcome impacts of PM_2.5_ across spatiotemporal domains particularly in the intermountain western United States where measurements from monitoring station data are sparse. Furthermore, these data may facilitate analyses of inversion impacts and local topography on exposure and health outcome studies.

## Abstract (summary)

**Table:** 

Measurement(s)	fine respirable suspended particulate matter
Technology Type(s)	air quality station
Factor Type(s)	MODIS AOD • Inversion Strength • Inversional Potential
Sample Characteristic - Environment	atmospheric zone
Sample Characteristic - Location	western United States • State of California • State of Oregon • State of Washington • State of Nevada • State of Utah • State of New Mexico • State of Arizona • State of Idaho • State of Montana • State of Colorado • State of Wyoming

## Background & Summary

Fine particulate matter (aerodynamic diameter <2.5 μm; PM_2.5_) is widely known to have significant adverse effects on human health^1–3^. While recent studies have shown air quality improving for the contiguous United States from the reduction of industrial and vehicular emissions^4,5^, air pollution in wildfire-prone areas, particularly in the mountain west region of the United States, has increased and is projected to further worsen due to climate-mediated increases in wildfire activity^6–8^. These communities impacted by wildfire smoke from nearby and distant wildfires experience high episodic exposures to PM_2.5_ with concentrations often exceeding 24-hour ambient air quality standards for extended periods^9,10^.

Of particular concern in western rural states is the scarcity of air quality monitoring stations, which provide the data needed to deliver accurate health warnings and predictions to the public. Air quality monitoring stations are often located to represent worst-case exposures for the largest concentration of people or sited to capture background exposure. For example, in 2021 there were 22 sites in the Montana network that monitored PM_2.5_ (16 = Population Exposures, 5 = Background Exposure, and 1 = Source Impact; https://www3.epa.gov/ttnamti1/files/ambient/pm25/qa/vol2sec06.pdf).

In the intermountain west, the sparsity of air quality monitoring stations is further complicated by the region’s complex terrain which likely contributes to significant heterogeneity in air pollution levels across communities^11^. Even in populous communities with fixed monitoring sites, the spatial variation can be high during the wildfire season due to inversion and drainage flow features common in the intermountain west. Many areas in the intermountain west also experience increased wintertime risk of poor air quality due to cold-air inversion, trapping air pollutants in mountain valleys where most towns and residents are located^12^. Regardless, it is unlikely that the single air monitor sites in many intermountain west communities provide an accurate representation of pollution exposure, as suggested by other urban area focused studies^13^. Thus, improved spatially resolved maps of surface PM_2.5_ accounting for variability in terrain are needed to enhance understanding of particulate matter impacts on public health during both the winter and wildfire season. Such maps would provide the spatial and topographically resolved data needed to identify fine scale PM_2.5_ effects on specific diseases, such as respiratory disease (e.g., influenza and wildfire season PM_2.5_^14^).

Previous studies have demonstrated that by combining ground observations with satellite and weather data, surface PM_2.5_ can be mapped with reasonably high accuracy. A wide range of modelling approaches have been evaluated, including machine learning algorithms^15,16^, spatio-temporal modelling and interpolation^17^, ensemble approaches^18^, linear regression^19,20^, and geographically weighted regression (GWR)^21^. Many of these models rely on satellite-based measurements of aerosol optical depth (AOD) data in combination with ground-based air quality observations. AOD measures the optical extinction in the air column. The spatial coverage of AOD data allows researchers the ability to fill in where EPA’s PM_2.5_ monitoring sites are lacking. However, AOD data have their limitations. AOD attempts to measure the mass of aerosols in the entire atmospheric column, which may not correlate with measurements at the surface. In many areas of the intermountain west snow and/or cloud cover will produce missing data, with some locations having no winter observations^22^. Likewise, the semi-arid western US MODIS AOD data are also unreliable due to high heterogeneous surface albedo^23^. Additional infilling techniques are needed when working across these areas and with MODIS data.

Despite extensive efforts to model surface PM_2.5_, few datasets are publicly available for use in air quality and public health studies^24^. Here, we describe the development of daily PM_2.5_ grids for the western United States. We use air quality observations from the EPA combined with weather data from global reanalysis, high resolution MODIS AOD data^20^ and static spatial covariates to produce daily grids at approximately 1-kilometer resolution from 2003–2020. The dataset we describe here improves upon previous daily PM_2.5_ models by providing one of the longest time series of daily surface PM_2.5_ and through the use of a terrain component designed specifically to capture the potential for increased surface PM_2.5_ in valleys under stable atmospheric conditions. This term provides a significant improvement in predictive accuracy year-round and particularly in winter when MODIS data are frequently unavailable.

## Methods

**Fig. 1:**
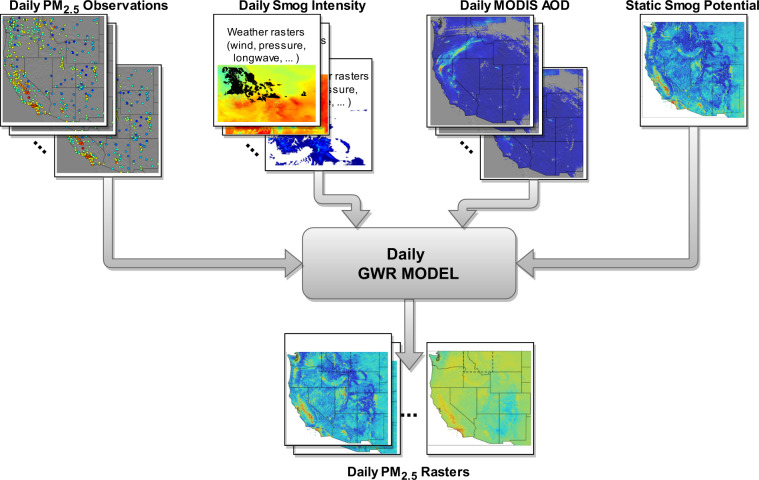
Schematic representation of the geographical weighted regression (GWR) modelling framework producing daily PM_2.5_ rasters (layers). Top row (left to right): Daily PM_2.5_ point observations from EPA monitoring stations^21^, daily smog intensity layers at 0.5-deg resolution comprised of various meteorological data from Climate Forecast System Reanalysis^23^, Daily MODIS data for Aerosol Optical Depth, and the static smog potential layer.

An overview of the data and methods is shown in Fig. 1.

### Study area

The study area consisted of the contiguous United States west of −102 degrees longitude, or the 11 western-most states. The study period began 2003-01-01 and ended 2020-12-14.

### Surface fine particulate matter measurements

**Fig. 2:**
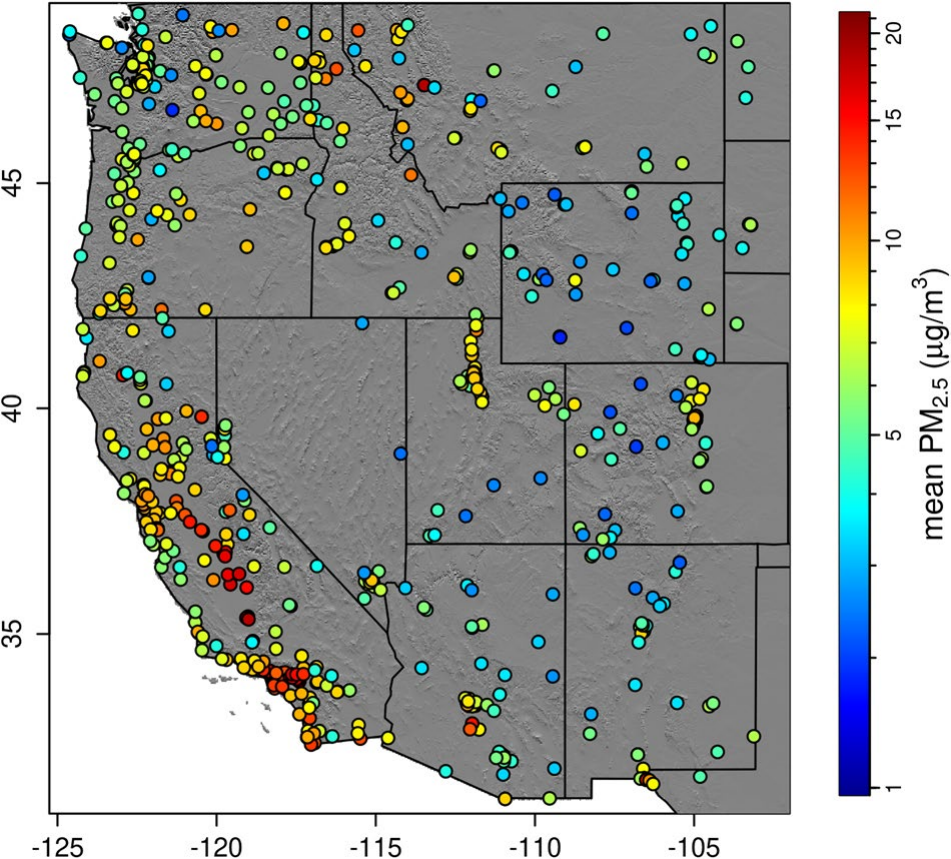
Mean (2003–2020) surface PM_2.5_ (μg/m^3^) at sampling locations.

We obtained surface PM_2.5_ measurements from the United States Environmental Protection Agency (EPA) Air quality monitoring system (AQS)^25^. This included 1,935,006 observations from 823 unique locations (Fig. 2). Hourly measurements were averaged over each day. The annual average daily count of observations ranged from 151 to 404, increasing each year, but with many stations recording every 3 days the count varied greatly from day to day. This variability was more pronounced at the beginning of the study.

### Predictor variables

#### Satellite aerosol optical depth

Satellite Aerosol Optical Depth (AOD) measures particulate matter in the atmosphere that is a column integrated optical measure of PM (specifically, total suspended particles) extinction in the atmosphere. We used daily MODIS MCD19A2 AOD at 1-km resolution as a predictor following many other studies^15–21^. AOD is noted to correlate well with wildfire smoke^26^, but is affected by high surface albedo in semi-arid regions of the US^23^, clouds and snow cover. In areas with persistent snow cover a pixel might be continuously infilled for up to 5 months, and on the date with the greatest cloud cover only 4% of the total pixels were available. To avoid cloud, snow and albedo artifacts we used a double masking approach described below.

#### Smog potential layer

We developed a single static 1-km resolution smog potential layer by combining a number of spatial predictor layers in a GBM modelling framework. The full list of predictors is given in Supplementary Table 1.

A suite of topographic indices were derived from the 30-m resolution National Elevation Dataset^27^ (NED) Digital Elevation Model (DEM) resampled to 1-km resolution. The primary terrain covariates we considered were local minima functions that measure the log of the vertical distance between a given point and the lowest elevation within a set radius. The radii considered ranged from 700-m to 50-km. We also considered the morphometric protection index^28^, a measure of terrain openness within a 2 km radius. As additional descriptors of terrain we included a suite of MODIS minimum and maximum land surface temperature (LST) monthly 2003–2012 normals (https://modis.gsfc.nasa.gov/data/dataprod/mod11.php).

To represent pollution sources, we retrieved a suite of 114 gridded PM_2.5_ pollution emission surrogates from the United States EPA SMOKE program^29^ at 4-km resolution (https://www.cmascenter.org/download/data.cfm) and resampled to the 1-km grid. In addition, we obtained gridded 60-m resolution population density data from the United States Census Bureau for 2010. The population density data were resampled to 1-km resolution then kernel smoothed using a 2.5-km radius gaussian kernel.

#### Daily smog intensity

For prediction of daily smog intensity, we considered gridded 0.5-degree resolution meteorological data from the Climate Forecast System Reanalysis (CFSR)^30^; including upward longwave surface radiation, 700-mb geopotential height, wind speed at 10-m above ground, mean relative humidity at 2-m above ground, maximum temperature at 2-m above ground, and boundary layer cloud cover. The geopotential height was standardized daily on a per-pixel basis by subtracting the mean for that day of the year (1979–2014) and dividing by the standard deviation.

#### Fire perimeters

We considered the area of actively burning wildfire in a post-hoc analysis of model performance. To estimate fire activity, we downloaded all available fire perimeter shapefiles from geomac^31^ and converted these to raster grids matching our 1-km grid. For each date, we summed the number of pixels within the available fire perimeters.

### Modelling

#### Daily surface PM_2.5_ prediction creation

Our general approach was to use daily Geographically Weighted Regression (GWR) to make gridded predictions of surface PM_2.5_ using 3 covariates: (1) daily AOD, (2) static smog potential, and (3) daily smog intensity. We also considered the interaction between smog potential and smog intensity. Our approach follows methods described by Holden *et al*.^32^ used for modelling spatiotemporal dynamics of nocturnal cold air drainage. All models were fitted in R version 4.1.3^33^, using the gbm^34^, dismo^35^, spgwr^36^, geoR^37^, gstat^38^ and raster^39^ packages plus custom code written by A.S. Our code is available with a worked example in Supplementary Material.

Daily average PM_2.5_ data were downloaded from the EPA AQS Transfer Network^25^. A log transformation was applied prior to modelling to correct for positive skewness of the PM_2.5_ observations: most observations are near zero (median = 5.8 ug/m^3^) but there were numerous extreme values as large as 818 ug/m^3^. Since our data contained zeros, 1 was added before applying the log transformation. Predictions in the log scale were back-transformed using the exponential function and subtracting 1. Log bias is mathematically expected in back-transformed log predictions, so we corrected for this using the Duan method^40^ on a daily basis, in which a linear model (LM) for observed surface PM_2.5_ as a function of predicted surface PM_2.5_ is fit with no intercept. The slope term of this LM is then used to multiply the back-transformed predictions. Since the back-transformation can yield negative values, we changed all negative values to zero. Similarly, we truncated all values greater than 1000 since the most extreme station observation was 818.

**Fig. 3:**
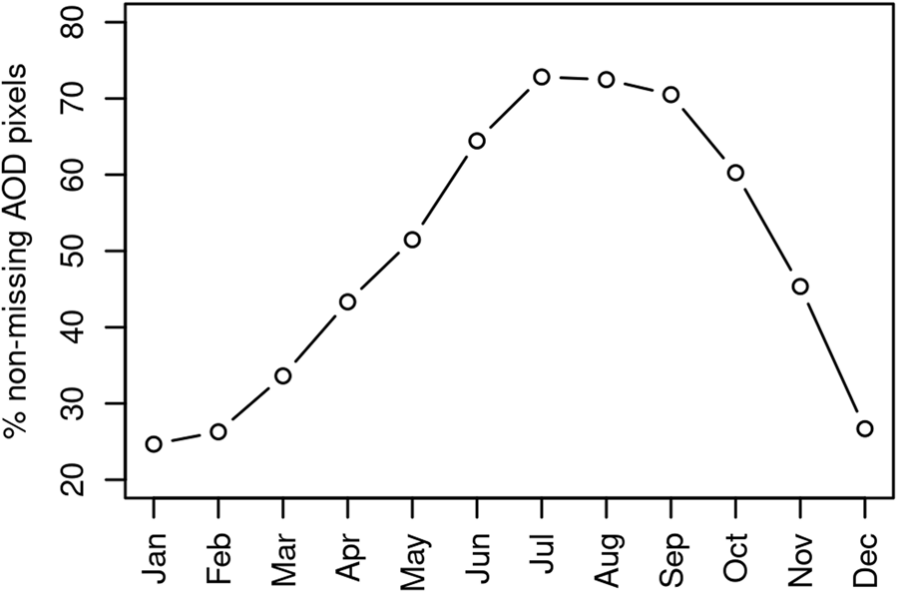
Percent of non-missing MODIS AOD pixels by month. AOD was relatively complete in summer and early fall which corresponds to wildfire season in the western United States^14^.

We retrieved 1-km resolution MODIS AOD data from the MODIS collection 6 MAIAC algorithm^22^. MODIS data have considerable contamination from snow and clouds, particularly in mountainous areas of the western United States, as well as high surface albedo in semi-arid regions of the US^23^. In addition, there are known issues with smoke being masked as cloud, which we were unable to address since it would require changes to the AOD retrieval algorithm^41^. We applied 3 methods for removing pixels which likely contained errors. First, we screened the data to include only high-quality pixels based on the quality assurance layer provided with the MODIS product. We then used data from the 250-m resolution 8-day MODIS Normalized Difference Vegetation Index (NDVI) provided by the Global Agricultural Monitoring System (GLAM)^42^. Previous work with these data^14^ showed that missing values from this product accurately indicated snow covered days. The snow mask derived from this source was buffered by 3 pixels to more conservatively eliminate erroneous values due to snow. Lastly, we developed a static mask for consistently bright areas due to highly reflective ground surface, such as may occur in salt pans and dry lake beds (https://lpdaac.usgs.gov/documents/110/MCD19_User_Guide_V6.pdf). This was based on a threshold of 100 applied to the 5^th^ percentile of fall (September through November) 2000–2020 values for each pixel. Following removal of missing or contaminated pixels, we used a simple spatial infilling approach to fill in missing pixels based on the nearest non-missing data values. Although crude, this was deemed adequate as we found our GWR models naturally down-weighted the infilled areas. Dates that were unavailable (n = 50) were temporally interpolated from the nearest previous and subsequent dates on a per-pixel basis. Proportion of non-missing data averaged by month is shown in Fig. 3.

**Fig. 4:**
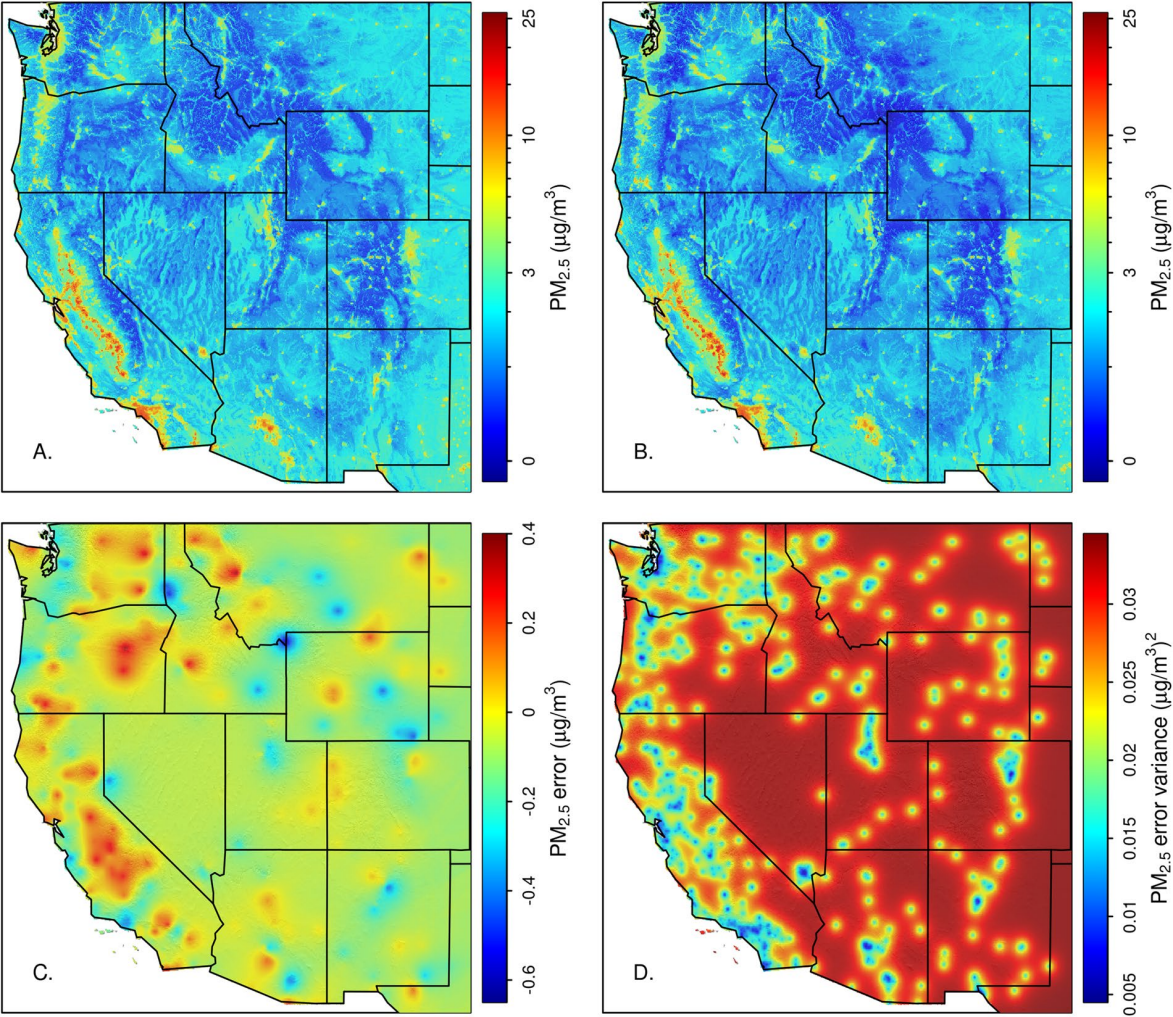
Smog potential raster predictions. A GBM machine learning model was used to predict mean station PM_2.5_ under stable winter conditions (**A**). Errors from this model were spatially interpolated using kriging (**C**). Red areas are where the model underpredicts smog potential while blue areas indicate overprediction. The GBM fit and kriged errors were combined to make the final smog potential raster (**B**). Panel D shows the kriging prediction variance.

We used an iterative approach to develop a static spatial model of smog potential and a dynamic model of daily smog intensity. Under stable atmospheric conditions, which occur most frequently during the winter months (December - February) in western North America, pollution can accumulate near its source, often settling into valleys and leaving upslope areas with relatively cleaner air^43^. Topography can greatly influence the degree of accumulation, with more enclosed valleys trapping more pollution. Our smog potential layer integrates pollution sources and topography to predict where surface PM_2.5_ accumulates under stable atmospheric conditions, while the smog intensity models when and where stable conditions occur. The following steps were used to create these layers:

1. We calculated mean winter PM_2.5_ at each station and modelled this as a function of local emissions and topography. Winter observations (Dec. 1 – Feb. 28) were used because the smog effect is strongest in winter and smoke-driven AOD effects are minimized. Locations with less than 100 observations were excluded. We used gradient boosting machines (GBM; also known as generalized boosted regression models or boosted regression trees)^44^ to fit this model, with spatially independent cross-validation (CV) to control for over-fitting. GBM is a machine learning method that fits a series of decision trees sequentially. The first tree is fit to the response variable and each subsequent tree is fit to the errors of the ensemble of trees leading up to that stage, with weighting used to emphasize difficult to fit observations. At each stage a new tree is chosen to minimize a loss function, which in our case is the sum of squared errors from a 10-fold cross validation. The CV groups were defined by dividing the study area based on k-means clustering of geographic coordinates. GBM models require 3 main parameters to be specified. The learning rate (also known as the shrinkage parameter) controls the relative contribution of each new tree. A larger learning rate causes the loss function to decrease more rapidly as trees are added. The tree complexity controls the number of branches of each tree, which is analogous to the order of interactions allowed. The third parameter is the number of trees. In our case we set tree complexity to 3 and chose a learning rate such that the loss function was minimized after 1000 to 2000 trees were added. All topographic and pollution source predictors described above and listed in Table S1 were put through an initial screening using Pearson’s correlation test, the remaining covariates were used in an initial GBM model. Model selection was accomplished by iteratively removing the predictor with the smallest contribution and choosing the smallest model to minimize CV log-likelihood, which is theoretically similar to AIC^45^, such that more complex models did not result in a substantial improvement. The final model was fit using all data and predictions were made at all sample locations
2. After the initial smog potential model was fit, we used a linear regression model to predict daily station PM_2.5_ using a suite of coarse-resolution (0.5-degree) weather covariates and their interactions with estimated smog potential. From this model we extracted the multiplier for smog potential by grouping the smog terms and considering only those regression coefficients including smog potential and its interactions. Equations 1–3 show how this was derived.
  1 $$\begin{array}{lll}{y}_{i} & = & {b}_{0}+{b}_{1}\ast ao{d}_{i}+{b}_{2}\ast smo{g}_{i}+{b}_{3}\ast ge{o}_{i}+{b}_{4}\ast longwav{e}_{i}+{b}_{5}\ast win{d}_{i}\\  &  & +{b}_{6}\ast smo{g}_{i}\ast ge{o}_{i}+{b}_{7}\ast smo{g}_{i}\ast longwav{e}_{i}+{b}_{8}\ast smo{g}_{i}\ast win{d}_{i}+\in \end{array}$$
  2 $$\begin{array}{ccc}{y}_{i} & = & {b}_{0}+{b}_{1}\ast ao{d}_{i}+{b}_{3}\ast ge{o}_{i}+{b}_{4}\ast longwav{e}_{i}+{b}_{5}\ast win{d}_{i}\\  &  & +({b}_{2}+{b}_{6}\ast ge{o}_{i}+{b}_{7}\ast longwav{e}_{i}+{b}_{8}\ast win{d}_{i})\ast smo{g}_{i}+\in \end{array}$$
  3 $$smog\_intensit{y}_{i}={b}_{2}+{b}_{6}\ast ge{o}_{i}+{b}_{7}\ast longwav{e}_{i}+{b}_{8}\ast win{d}_{i}$$
3. A preliminary smog intensity was calculated by predicting Eq. (3) to all times and locations.
4. We developed a refined estimate of station smog potential by averaging surface PM2.5 under a set of winter days (Dec. 1 – Feb. 28) within a constrained range of preliminary smog intensity values. Stations with fewer than 100 observations meeting this criteria were removed. Mean surface PM2.5 under these standardized conditions was again modelled using GBM as in step 1, giving a final model for smog potential. Predictions from this model were made over the 1-km grid.
5. A final linear model was fit using the refined smog potential model and its interactions with weather covariates. The smog intensity derived from this linear model was used as a predictor for daily surface PM2.5 as in step 2. Leave one out cross-validation (LOOCV) yielded an R^2^ of 0.73.
6. The errors in the smog potential layer were examined for spatial dependency. Variogram modelling using an exponential function estimated autocorrelation out to an effective range of 156-km. These errors could be due to atmospheric effects or pollution sources not accounted for in our model of smog potential. Addition of the kriged error term to the GBM fits increased LOOCV R^2^ at sample locations to 0.76. The GBM errors were kriged over the 1-km grid and added to the GBM fit to produce a final model of static smog potential. The initial smog potential fit, kriged error surface and final model are shown in Fig. 4.

Geographically weighted regression (GWR)^46^ was used to interpolate daily station PM_2.5_ using the smog potential, smog intensity, interaction between smog potential and smog intensity and AOD as predictors. This technique fits a unique weighted linear regression model at a set of user defined points, with weights based on the geographic distance from each point to the location of PM_2.5_ observations. GWR is an ideal technique to use when the relationships between the response and predictors are non-stationary in space, such as other studies have noted in the relationship between AOD and PM_2.5_. Since the AOD and PM_2.5_ relationship is known to vary seasonally^14^, we fit a unique GWR model daily. To define weights, we chose a gaussian kernel with an adaptive bandwidth selected to include a fixed proportion of data points. We found that the optimal proportion varied with the number of daily observations, so we used a value of 10% for days with *n* > 200 observations, 20% for 100 < *n* < 200, and 50% for *n* < 100.

The daily GWR fits gave an additional advantage in dealing with the large swaths of missing values in satellite AOD. The large infilled gaps in the AOD covariate cause its value as a predictor to vary in space and time. Daily GWRs allow this predictor to be down-weighted when and where its information content is low.

To evaluate the predictive performance of our model we performed a leave one out cross-validation (LOOCV)^47^. In this scheme, for each day, a series of GWR models are fit with a single observation withheld. We also refit out static smog model with each point withheld. Predictions to the withheld locations are retained for model validation. Thus, a separate GWR was fit to each and every PM_2.5_ observation. LOOCV provides a measure of predictive accuracy analogous to considering an entirely new location.

In addition to fitting the full model, which includes both AOD and smog terms, we fit an intercept-only null model, an AOD-only model, and a smog-only model. This allowed for a comparison of the relative strength of the predictors.

We made our GWR predictions to a coarser 10-km grid to ease the computation burden. The set of regression coefficients estimated at each point of the 10 km grid were resampled to the 1-km grid using bilinear interpolation, then multiplied by the 1-km covariates to give 1-km resolution predictions of PM_2.5_. We found this gave a negligible loss in predictive accuracy because the GWR coefficients varied spatially on a scale much larger than 10-km.

## Data Records

Table 1 lists the names and descriptions of the datafiles that are available on figshare^48^. We provide annual files in NetCDF format containing daily 1-km resolution surface PM_2.5_ estimates. We also provide the static 1-km resolution smog potential layer. Additional input data sources are publicly available and accessible online. R code used in our analysis is also provided.

**Table 1:** Data files provided at figshare^48^.

Name of files	Years of Data	Variables
PM25_west_pred_[year].nc	2003–2020	Estimated Surface PM2.5
Smog_potential_1km.tif	Static layer	Probability (0–1)

## Technical Validation

### Performance of static smog prediction model

Table 2 shows correlation coefficients between the coarse resolution CFSR meteorological variables and daily winter PM_2.5_ at all locations, as in step 1 of the methodology for developing the smog layers. These show that mean surface PM_2.5_ is higher under stable conditions defined by low wind, high pressure, low humidity, strong upward longwave radiation flux, and low cloud cover. MODIS AOD and surface Tmax are relatively poor predictors of winter PM_2.5_. The top 5 predictors were used in a linear model to predict daily smog intensity.

**Table 2:** Correlation of coarse resolution gridded CFSR meteorological variables and MODIS AOD to daily winter PM_2.5_ across all locations.

Reanalysis variable	Correlation coefficient
10 m Wind	−0.217
Standardized Geopotential Height	0.211
2 m relative humidity	−0.185
Upward surface longwave flux	0.182
Boundary layer cloud cover	−0.179
2 m Tmax	0.080

This shows that mean surface PM_2.5_ is higher under stable conditions defined by low wind, high pressure, low relative humidity, high cloud cover, etc.

Table 3 shows the correlation between the top 11 spatial predictors and mean surface PM_2.5_ of stations under stable conditions as defined by the daily smog intensity model. Population density is the strongest predictor but the metrics of slope position are also strong as indicated by the high correlation coefficients for the local minima functions, which measure the vertical distance between a given location and the lowest point within a defined radius. These predictors were used in a GBM to produce gridded estimates of smog potential. The raster map was able to explain 78% of the variation in estimated smog potential. As a final step we kriged the prediction errors over our 1-km grid using an exponential variogram function with an effective range of 156-km. This gave a final map that explained 89% of the variation in estimated smog potential. We also performed a LOOCV on the GBM model and kriging step. For each iteration we withheld a single point to fit the GBM and perform the error kriging. This gave *R*^2^ values of 0.755 and 0.719 for the predictions with and without the kriged error term, respectively.

**Table 3:** Correlation of spatial predictors to mean station PM_2.5_ under stable conditions.

Spatial predictor	Correlation coefficient
Population density	0.722
700 m local minima fct.	−0.605
10 km local minima fct.	−0.561
15 km local minima fct	−0.559
20 km local minima fct	−0.551
2500 m local minima fct.	−0.543
25 km local minima fct.	−0.543
5 km local minima fct.	−0.499
50 km local minima fct.	−0.499
industrial land	0.345
Long-haul trucking	0.340

These predictors were used to inform the model of static smog potential. Population density was consistently the best predictor of surface PM_2.5_ pollution under such conditions.

### Performance metrics on testing data

**Fig. 5:**
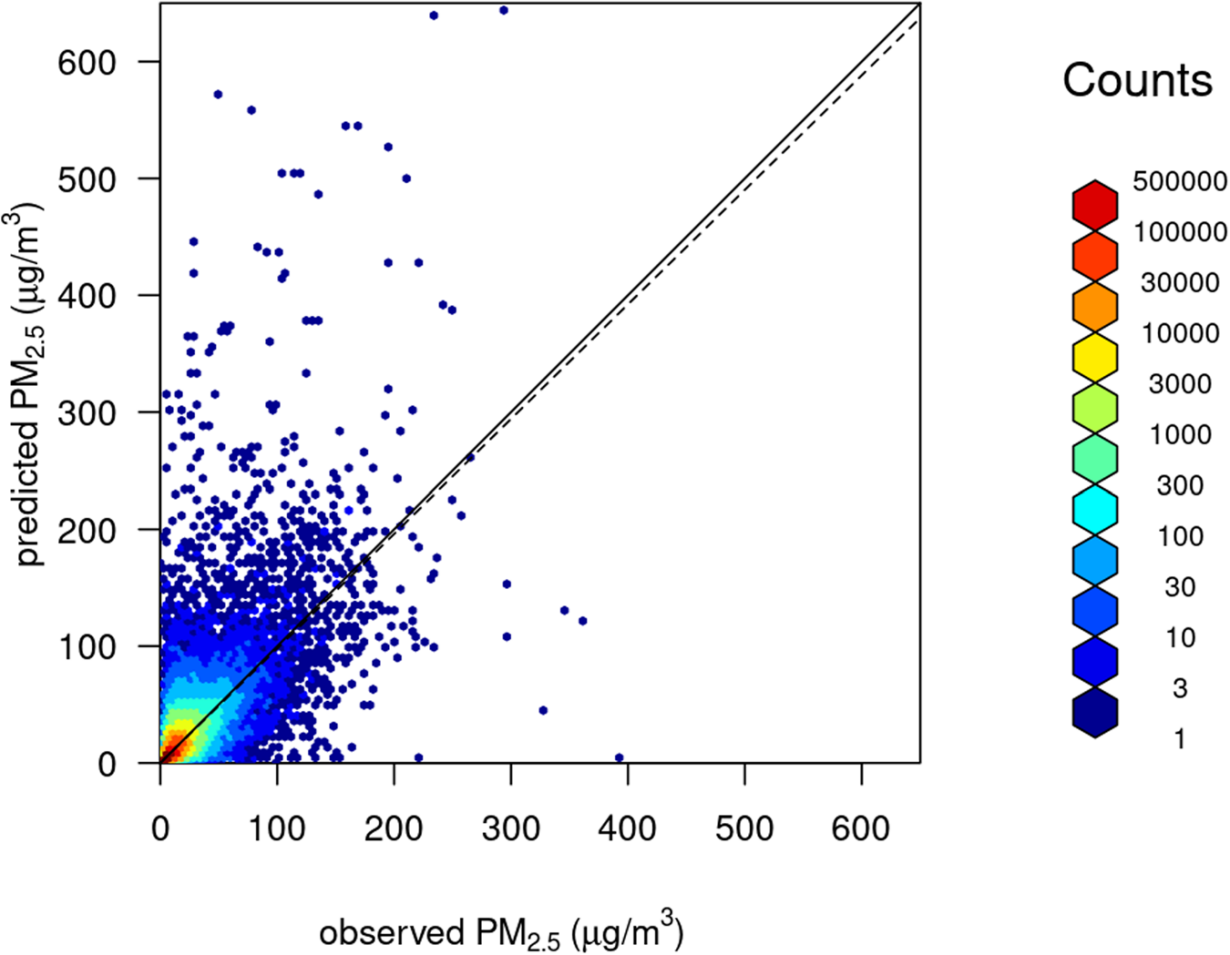
Scatterplot showing observed vs. predicted surface PM_2.5_ for the full model from a LOOCV representing completely independent validation. A regression line forced through the origin is show as a dashed line gave a slope of 0.98 with a standard error of 0.0004. The solid line shows 1:1. *R*^2^ = 0.554.

**Fig. 6:**
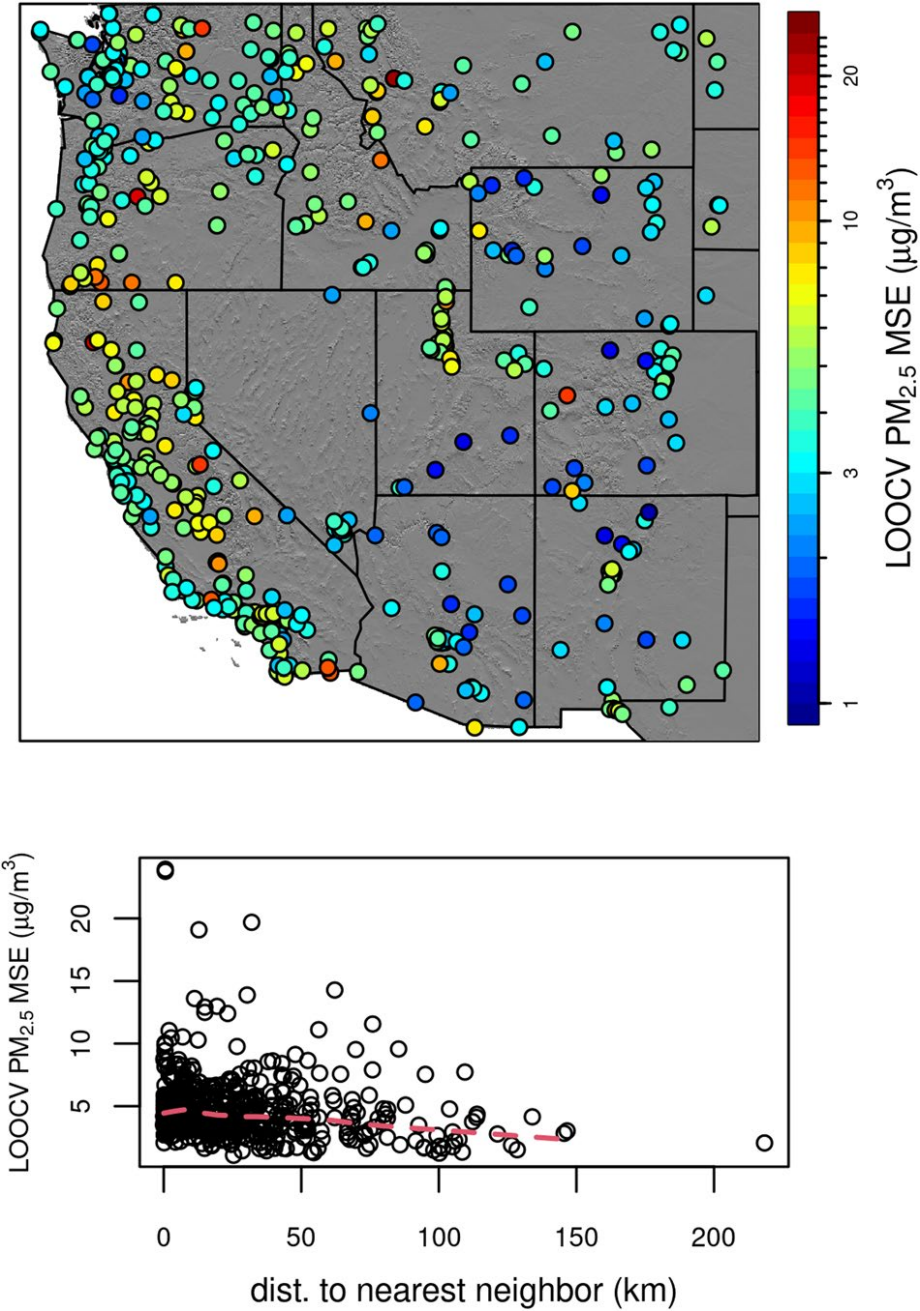
Map of RMSE from the LOOCV representing independent validation (top panel). Bottom panel shows distance between each station and RMSE.

Table 4 shows the performance metrics (RMSE, MAE and *R*^2^) resulting from the LOOCV validation of our full model and the 3 reduced models. The results from our full model are comparable to similar studies also using stringent validation on spatially independent data (Reid *et al*. 2021). Figure 5 shows a scatterplot of observed versus LOOCV predicted values. This shows that our model tends to underpredict the largest extreme values. A regression of predicted on observed values and forced through the origin yielded a slope of 1.00 (SE = 0.0004). Figure 6 shows the spatial representation of the RMSE with a scatterplot against the distance between each stations, showing larger distances with smaller error structure.

**Table 4:** Leave-one-out summary statistics (*R*^2^, Mean Absolute Error (MAE), Root Mean Square Error (RMSE)) from the null, AOD, smog and full (smog + AOD) geographically weighted regression models for the years 2003–2020.

	*R*^2^	MAE	RMSE
Null	0.462	3.64	7.21
AOD	0.546	3.47	6.62
Smog only	0.578	2.82	6.38
Full	0.646	2.72	5.84

Note that the RMSE is approximately the same as the median value for this study.

Note that RMSE is highly correlated with average PM2.5 (*r* = 0.57), since low PM_2.5_ values limit the magnitude of errors.

**Fig. 7:**
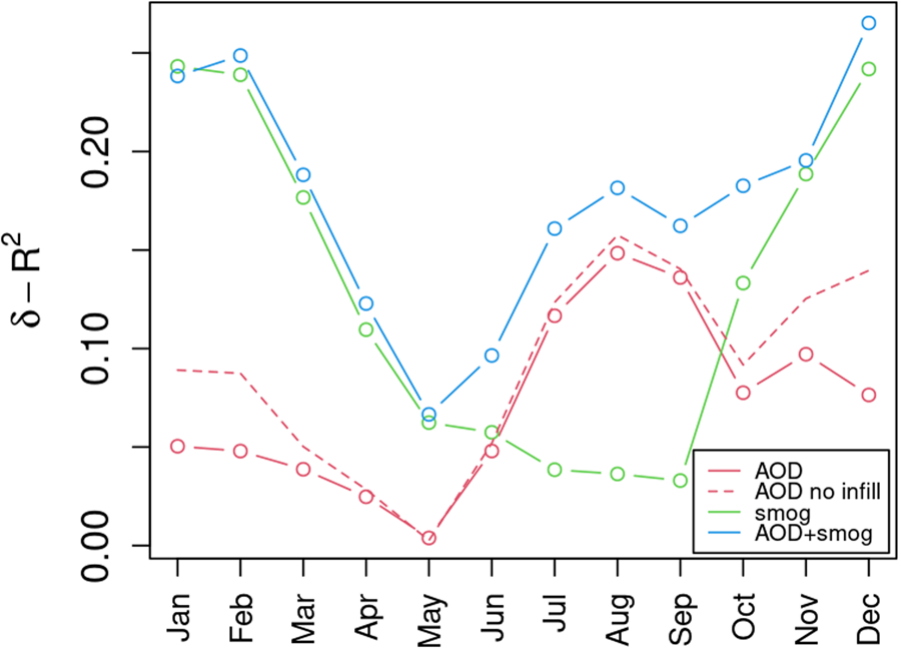
Performance of models relative to the intercept-only GWR null model. MODIS AOD (red lines) provides a modest improvement in fit during fire season (July - September) while the smog covariate (green line) performs well in the winter (Oct – Mar). The combined model (blue line) shows the improvements with both AOD in the summer months and smog in the winter months.

**Fig. 8:**
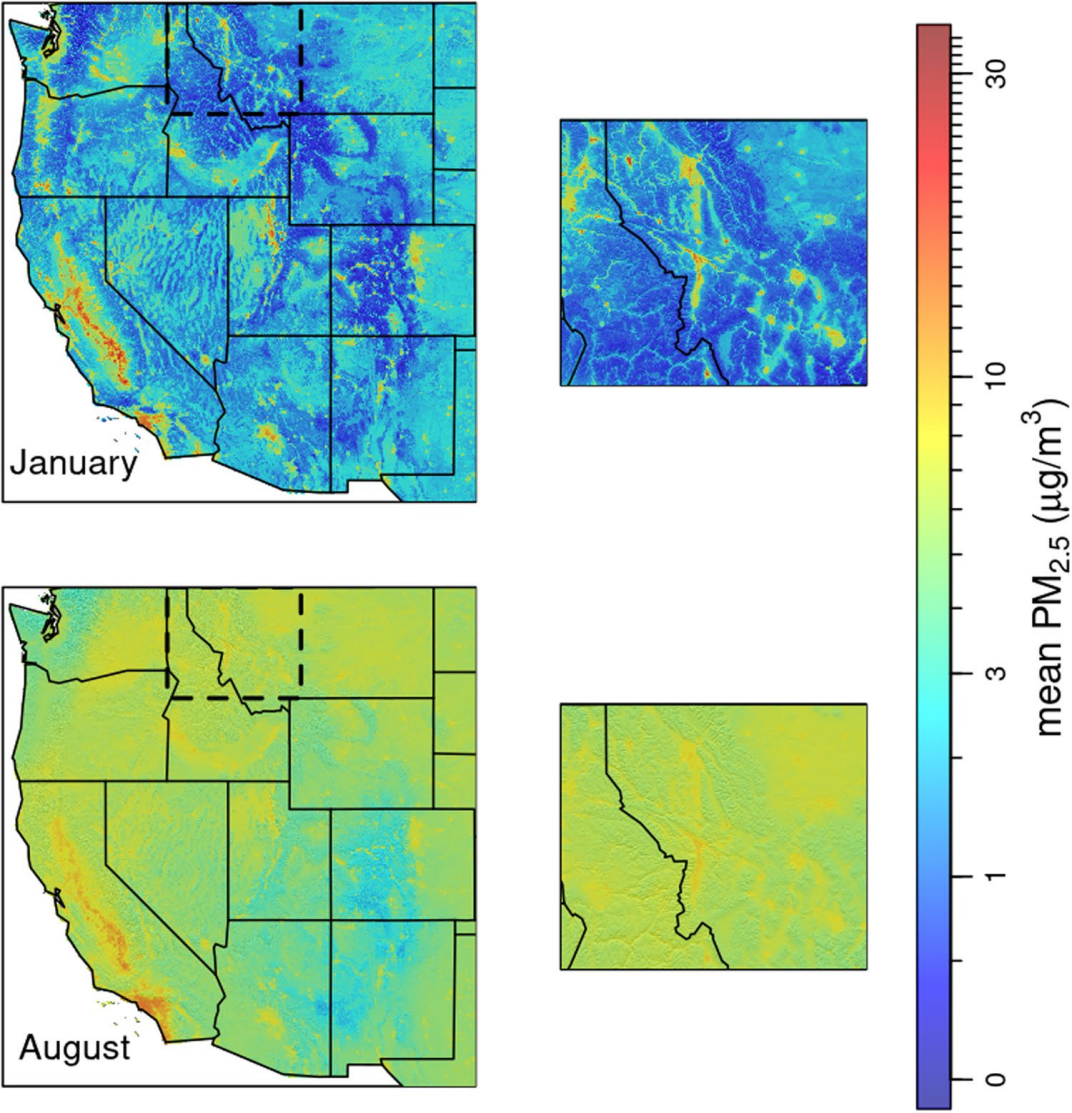
Surface PM_2.5_ normals (2003–2020) for January and August. The insets show western Montana and northern Idaho. Summer PM_2.5_ is widely distributed with little regard to topography while winter PM_2.5_ is more localized to population centers and often accumulates in confined valleys.

Comparison of the full and reduced models yielded results that support the hypothesis that wildfire smoke is the dominant source of PM_2.5_ in the summer and early fall, while smog is dominant in winter. Figure 7 shows the difference in *R*^2^ by month between full, AOD-only and smog-only models versus the null model, which was an intercept-only GWR. The smog sub-model contributes strongly in the winter and less so in the summer and early fall. This is not surprising since the stable conditions which allow smog to accumulate occur predominantly in the winter months over western North America. In contrast, the AOD-only model outperforms the smog-only model in July, August and September. This is also not surprising as these are the months considered fire season in western North America. Figure 8 shows raster odell for January and August, calculated by averaging all raster maps for those months. It shows how our model predicts population density and topography to dominate in winter months while during the summer PM_2.5_ pollution is more widely distributed, presumably due to the dispersed effect of wildfire smoke.

**Fig. 9:**
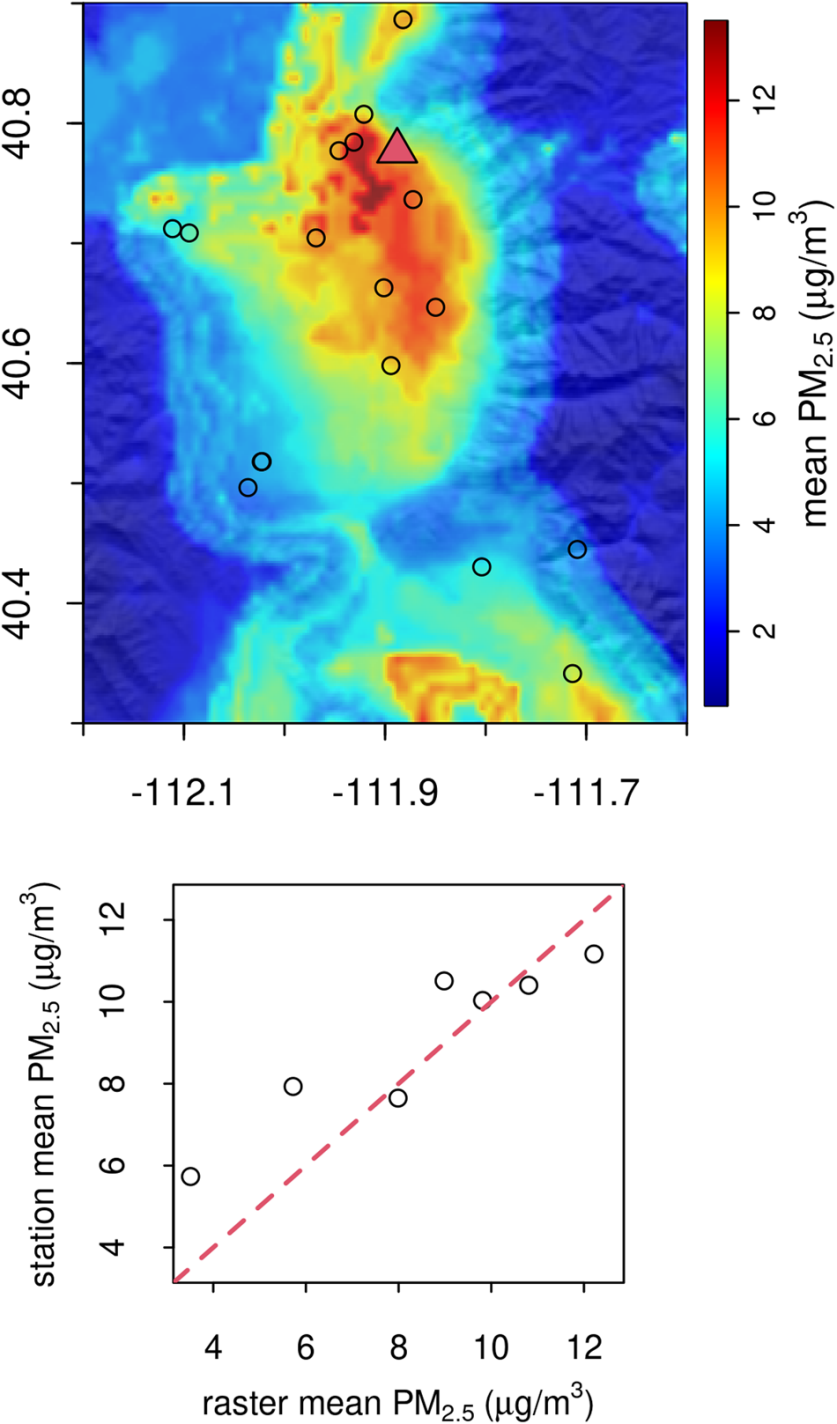
Example 2019 winter mean PM_2.5_ for Salt Lake City, Utah, USA (red triangle) area. Open circles represent the monitoring stations. Bottom panel shows the relationship between the monitoring stations and the modeled PM_2.5_ values.

Since AOD is hampered by missing values during the winter months, we also calculated *R*^2^ for the AOD-only model for only those observations for which non-infilled AOD was available. This increased the apparent performance of the AOD-only model during the winter months, but a true comparison is difficult since the subset of valid AOD winter observations generally come from the southwest portion of our study area where snow and cloudy conditions are less predominant. Figure 9 gives an example for the Salt Lake City, Utah, USA, area for the observation station values correlated to the modelled raster mean PM_2.5_ (μg/m^3^; *R*^2^ = 0.554).

**Fig. 10:**
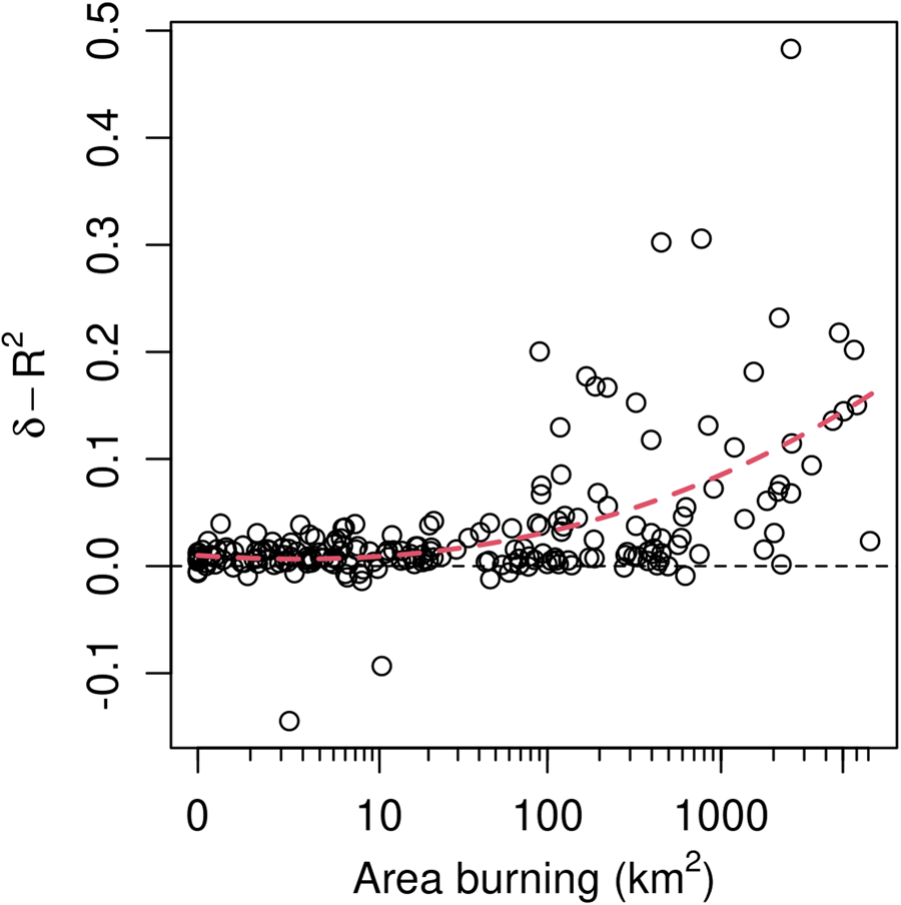
Improvement in *R*^2^ for AOD-only model relative to the null model versus area of actively burning wildfire. Each point represents a single month of data over all stations. Red dashed line shows a cubic regression fit. MODIS AOD provides an improvement in fit during active fire season.

The relationship between AOD predictive performance and wildfire smoke is further borne out by comparing AOD model performance versus wildfire activity. Figure 10 shows the relative difference in monthly model performance between the AOD-only and null models with respect to the area of actively burning wildfire for those same months. We see that the AOD covariate becomes increasingly important when area burning exceeds 100 km^2^. This implies that AOD is able to effectively delineate the spatial pattern of wildfire smoke.

In summary, we feel our model provides for realistic predictions of PM_2.5_ pollution across areas of the western United States where measurements are sparse. We show that topography and pollution sources can be used to predict the distribution of PM_2.5_ under stable meteorological conditions, which complements the well-documented ability of satellite-based AOD to predict PM_2.5_ driven by wildfire. We hope the public availability of these data will prove useful to researchers studying the health effects of PM_2.5_ pollution.

## Supplementary information

Supplementary information

## Data Availability

All code used for downloading and processing the data used in this project, including the modelling and technical validation code, may be accessed at figshare^48^. To ensure that our work is reproducible, all code is written in open-source languages.

## References

[1] US EPA (U.S. Environmental Protection Agency). Integrated Science Assessment (ISA) For Particulate Matter (Final Report). EPA/600/R-08/139F.Washington, DC: U.S. EPA (2009). 36630543

[2] Anderson JO, Thundiyil JG, Stolbach A (2012). Clearing the air: a review of the effects of particulate matter air pollution on human health. J. Med. Toxicol..

[3] Kim K-H, Kabir E, Kabir S (2015). A review on the human health impact of airborne particulate matter. Environ Int.

[4] McClure CD, Jaffe DA (2018). US particulate matter air quality improves except in wildfire-prone areas. PNAS..

[5] O’Dell K (2019). The contribution of wildland-fire smoke to US PM2.5 and its influence on recent trends. Environ. Sci. Technol..

[6] Yue X (2013). Ensemble projections of wildfire activity and carbonaceous aerosol concentrations over the western United States in the mid-21st century. Atmos. Environ..

[7] Liu JC (2016). Particulate air pollution from wildfires in the Western US under climate change. Clim. Change.

[8] Ford, B. *et al*. Future fire impacts on smoke concentrations, visibility, and health in the contiguous United States. *GeoHealth* **2** (2018). 10.1029/2018GH000144 PMC7038896 32159016

[9] Liu JC, Pereira G, Uhl SA, Bravo MA, Bell ML (2015). A systematic review of the physical health impacts from non-occupational exposure to wildfire smoke. Environ Res.

[10] Orr A, Migliaccio C, Buford M, Ballou S, Migliaccio CT (2020). Sustained Effects on Lung Function in Community Members Following Exposure to Hazardous PM2.5 Levels from Wildfire Smoke. Toxics.

[11] Armstrong BG (1998). Effect of measurement error on epidemiological studies of environmental and occupational exposures. Occup. Environ. Med..

[12] Ward T, Lange T (2010). The impact of wood smoke on ambient PM2.5 in northern Rocky Mountain valley communities. Environ Pollut..

[13] Tunno BJ (2016). Spatial patterning in PM2.5 constituents under an inversion-focused sampling design across an urban area of complex terrain. J. Exposure Sci. Environ. Epidemiol..

[14] Landguth EL (2020). The delayed effect of wildfire season particulate matter on subsequent influenza season in a mountain west region of the USA. Environment International.

[15] Hu X (2017). Estimating PM2.5 Concentrations in the Conterminous United States Using the Random Forest Approach. Environ. Sci. Technol..

[16] Park Y (2020). Estimating PM2.5 concentration of the conterminous United States via interpretable convolutional neural networks. Environ. Pollut..

[17] Hu H (2019). Satellite-based high-resolution mapping of ground-level PM2.5 concentrations over East China using a spatiotemporal regression kriging model. Sci. Total Environ..

[18] Di Q (2019). An ensemble-based model of PM2.5 concentration across the contiguous United States with high spatiotemporal resolution. Environ. Int..

[19] Chu DA (2006). Analysis of the relationship between MODIS aerosol optical depth and PM2.5 in the summertime US. Remote Sensing of Aerosol and Chemical Gases, Model Simulation/Assimilation, and Applications to Air Quality.

[20] Ma Z, Hu X, Huang L, Bi J, Liu Y (2014). Estimating ground-level PM2. 5 in China using satellite remote sensing. Environmental science & technology.

[21] Song W, Jia H, Huang J, Zhang Y (2014). A satellite-based geographically weighted regression model for regional PM2.5 estimation over the Pearl River Delta region in China. Remote Sensing of Environment.

[22] Lyapustrin A, Wang Y, Korkin S, Huang D (2018). MODIS collection 6 MAIAC algorithm. Atmos. Meas. Tech..

[23] Loría-Salazar SM, Holmes HA, Arnott WP, Bernard JC, Moosmuller H (2016). Evaluation of MODIS Columnar Aerosol Retrievals Using AERONET in Semi-Arid Nevada and California, U.S.A during the Summer of 2012. Atm. Env..

[24] Reid CE (2021). Daily PM2.5 concentration estimates by county, ZIP code, and census tract in 11 western states 2008–2018. Sci Data.

[25] Technology Transfer Network (TTN) Air Quality System (AQS); U.S. Environmental Protection Agency; available at www.epa.gov/aqs (accessed January 2021).

[26] Mirzaei M, Bertazzon S, Couloigner I, Farjad B, Ngom R (2020). Estimation of local daily PM2. 5 concentration during wildfire episodes: integrating MODIS AOD with multivariate linear mixed effect (LME) models. Air Quality, Atmosphere & Health.

[27] Gesch D (2002). The national elevation dataset. Photogrammetric engineering and remote sensing.

[28] Yokoyama R, Shirasawa M, Pike RJ (2002). Visualizing topography by openness: a new application of image processing to digital elevation models. Photogrammetric engineering and remote sensing.

[29] Houyoux MR, Vukovich JM (1999). Updates to the Sparse Matrix Operator Kernel Emissions (SMOKE) modelling system and integration with Models-3. The Emission Inventory: Regional Strategies for the Future.

[30] Saha S (2010). The NCEP climate forecast system reanalysis. Bulletin of the American Meteorological Society.

[31] Walters, S. P., Schneider, N. J. & Guthrie, J. D. Geospatial Multi-Agency Coordination (GeoMAC) Wildland Fire Perimeters, 2008. *US Geological Survey Data Series* **612**(6) (2011).

[32] Holden ZA (2016). Development of high-resolution (250 m) historical daily gridded air temperature data using reanalysis and distributed sensor networks for the US northern Rocky Mountains. International Journal of Climatology.

[33] R Core Team. R: A language and environment for statistical computing. R Foundation for Statistical Computing, Vienna, Austria. https://www.R-project.org/ (2022).

[34] Greenwell, B., Boehmke, B. & Cunningham, J. GBM Developers. gbm: Generalized Boosted Regression Models. R package version 2.1.8 https://CRAN.R-project.org/package=gbm (2020).

[35] Hijmans, R. J., Phillips, S., Leathwick, J. & Elith, J. dismo: Species Distribution Modeling. R package version 1.3–5. https://CRAN.R-project.org/package=dismo (2021).

[36] Bivand, R., Yu, D. spgwr: Geographically Weighted Regression. R package version 0.6-35 https://CRAN.R-project.org/package=spgwr (2022).

[37] Ribeiro, P. J. Jr., Diggle, P. J., Schlather, M., Bivand, R., Ripley, B. geoR: Analysis of Geostatistical Data. R package version 1.8.1 https://CRAN.R-project.org/package=geoR (2020).

[38] Gräler B, Pebesma E, Heuvelink G (2016). Spatio-Temporal Interpolation using gstat. The R Journal.

[39] Hijmans, R. J. raster: Geographic Data Analysis and Modeling. R package version 3.5–15. https://CRAN.R-project.org/package=raster (2022).

[40] Duan N (1983). Smearing estimate: A non-parametric retransformation method. J. Amer. Statistical Society..

[41] (2015). NASA Level 1 and Atmosphere Archive and Distribution System.

[42] Becker-Reshef I (2010). Monitoring global croplands with coarse resolution earth observations: The global agricultural monitoring project. Remote Sensing.

[43] Whiteman CD, Hoch SW, Horel JD, Charland A (2014). Relationship between particulate air pollution and meteorological variables in Utah’s Salt Lake Valley. Atmospheric Environment.

[44] Friedman JH (2001). Greedy function approximation: A gradient boosting machine. Ann. Statist..

[45] Hauenstein S, Wood SN, Dormann CF (2018). Computing AIC for black-box models using generalized degrees of freedom: A comparison with cross-validation. Communications in Statistics-Simulation and Computation.

[46] Brunsdon C, Fotheringham AS, Charlton ME (1996). Geographically weighted regression: a method for exploring spatial nonstationarity. Geographical analysis.

[47] Efron, B. The Jackknife, the Bootstrap and other resampling plans. In *CBMS-NSF regional conference series in applied mathematics 1982*. Philadelphia, PA: Society for Industrial and Applied Mathematics (SIAM) (1982).

[48] Swanson A (2022). figshare.

